# Effect of Exogenous Melatonin on the Development of Mice Ovarian Follicles and Follicular Angiogenesis

**DOI:** 10.3390/ijms222011262

**Published:** 2021-10-19

**Authors:** Jingli Tao, Liangliang Zhang, Xuan Zhang, Yuanyuan Chen, Qianqian Chen, Ming Shen, Honglin Liu, Shoulong Deng

**Affiliations:** 1 College of Animal Science and Technology, Nanjing Agricultural University, Nanjing 210095, China; taojingli@njau.edu.cn (J.T.); 2018105016@njau.edu.cn (L.Z.); 2018105015@njau.edu.cn (X.Z.); 2019105022@njau.edu.cn (Y.C.); 2021105017@njau.edu.cn (Q.C.); shenm2015@njau.edu.cn (M.S.); 2 Institute of Laboratory Animal Sciences, Chinese Academy of Medical Sciences and Comparative Medicine Center, Peking Union Medical College, Beijing 100021, China

**Keywords:** melatonin, secondary follicles, angiogenesis, follicle-stimulating hormone (FSH), antioxidant enzymes

## Abstract

In mammalian, the periodic growth and development of ovarian follicles constitutes the physiological basis of female estrus and ovulation. Concomitantly, follicular angiogenesis exerts a pivotal role in the growth of ovarian follicles. Melatonin (N-acetyl-5-methoxytryptamine, Mel), exists in follicle fluid, was suggested to affect the development of follicles and angiogenesis. This research was conducted to investigate the effects and mechanisms of Mel on the development of ovarian follicles and its angiogenesis. In total, 40 ICR mice at age of 3 weeks were allocated into four groups at liberty: control, Mel, FSH and FSH + Mel for a 12-day trial. Ovaries were collected at 8:00 a.m. on Day 13 for detecting the development of ovarian follicles and angiogenesis. Results indicated that Mel promoted the development of ovarian follicles of 50–250 μm (secondary follicles) and periphery angiogenesis, while FSH remarkably increased the number of antral follicles and periphery angiogenesis. Mechanically, Mel and FSH may regulate the expression of VEGF and antioxidant enzymes in different follicular stages. In conclusion, Mel primarily acted on the secondary follicles, while FSH mainly promoted the development of antral follicles. They both conduced to related periphery angiogenesis by increasing the expression of VEGF. These findings may provide new targets for the regulating of follicular development.

## 1. Introduction

Follicular development is the physiological basis of female estrus and ovulation. In mammalian, more than 99% of the ovarian follicles undergo atresia during development [1]. Once the follicle begins to enter the developmental stage, its fate cannot be reversed, and either becomes a dominant follicle and releases a mature oocyte, or it moves towards atresia [2]. Generally, growing follicles were classified as primary follicles, secondary follicles, and tertiary follicles. Tertiary follicles include early antral follicles, late antral follicles and preovulatory follicles. Among them, the primary and secondary follicles belong to the gonadotropin-independent stage; while in the tertiary follicle, the follicle acquires the sensitivity to gonadotropins, thereby being selected to grow towards the preovulatory follicle, which finally release oocyte under the stimulation of luteinizing hormone (LH) [3].

Follicular blood vessels play a pivotal role in ovarian follicular development. Follicular capillaries provide essential nutrients, hormones and oxygen required for folliculogenesis [4]. The onset and establishment of the follicle vasculature takes place early during follicular development [5], and increased number of blood vessels is generated as follicles grow. Developing follicles have a vascular network in the theca layers outside the membrane propria. These blood vessels could not penetrate into the follicular membrane, but will gradually form branches to provide sufficient nutrients for follicular development. Therefore, a higher-level vascular network is essential for the preferential development of dominant follicles [6,7,8].

The development of blood vessels is regulated by some follicle-derived factors. In particular, vascular endothelial growth factors (VEGFs) and its receptors play a vital role in the process of follicular angiogenesis [9]. In mammals, vascular endothelial growth factor has five members: VEGFA, VEGFB, VEGFC, VEGFD and placental growth factor (PGF) [3]. Studies have found that primordial follicles and atretic follicles have no VEGF expression [10,11]. The immunostaining of VEGF in granulosa cells was observed from small antral follicles to preovulatory follicles, whereas VEGF expression in thecal cells was detected from medium-sized to preovulatory follicles. The abundance of VEGF was gradually increased as follicular growth [12]. VEGF secreted by granulosa cells and thecal cells promotes the follicular micro-angiogenesis, which facilitates accessibility of follicle-stimulating hormone (FSH) and luteinizing hormone (LH) to support the development of antral follicles. VEGF also increases the permeability of micro-vessels in the theca layers, leading to plasma extravasation and follicular fluid accumulation, suggesting that VEGF is related to follicular selection and development [13]. On the other hand, gonadotropins in turn stimulate the expression of VEGF in in ovarian follicles. As reported, increased mRNA level of VEGF was determined in granulosa cells of secondary and tertiary follicles, which rely on FSH stimulation for development [14]. Further studies demonstrated that the pro-angiogenetic effects of FSH on peripheral follicles is mediated in part through up-regulating VEGF expression [15,16]. 

Due to the avascular environment within ovarian follicles, granulosa cells are believed to live under hypoxia. During follicular development, the growing sizes of follicles create a progressively more hypoxic status in granulosa cells [17]. Hypoxia-inducible factor-1 (HIF-1), a highly specific nuclear transcription factor, functions as a key regulator of cellular response to microenvironmental oxygen partial pressure [18]. HIF-1 consists of a constitutively expressed subunit HIF-1β and an oxygen-regulated subunit HIF-1α [19]. HIF-1α is easily degraded under normoxia. In hypoxia, HIF-1α accumulates, and then enters in nucleus and combines with HIF-1β to form a complex to interact with the hypoxia response element (HRE) in the target gene for initiating transcription and regulating multiple physiological and pathological processes such as angiogenesis, cell proliferation and apoptosis and glucose metabolism [20,21,22]. In ovarian follicles, HIF-1α has been identified as the main transcriptional regulator of VEGFA-dependent angiogenesis [23,24]. 

Melatonin (N-acetyl-5-methoxytryptamine, Mel) has a wide range of biological effects, such as regulating the circadian clock, reproduction, immunity and antioxidation [25]. In the regulation of the reproductive system, Mel, synthesized and secreted by the pineal gland, can act on hypothalamic gonadotropin releasing hormone (GnRH) neurons through cerebrospinal fluid or blood circulation, and affect the hypothalamus-pituitary-the gonadal axis to regulate the reproductive activities of mammals [26,27]. As a potent antioxidant, the melatonin in follicular fluid could effectively scavenge excessive ROS (reactive oxygen species) produced during folliculogenesis, thereby mitigating the adverse effects of oxidative damage on follicular development [28,29,30]. Mel also modulates angiogenesis [31]. Under different physiological and pathological conditions, Mel has different effects on new blood vessels. Mel inhibits the neovascularization in tissues of tumors and age-related ocular diseases [32,33]. Contrariwise, it promotes angiogenesis in gastric ulcers and skin lesions [34,35]. However, during the development of follicles, the effect of Mel on angiogenesis around the follicle is rarely studied. 

Heme oxygenase-1 (HO-1), having a vital role in the catabolism of heme, is one of the downstream antioxidative enzyme of Mel. By catalyzing the degradation of heme, HO-1 yields equimolar amounts of biliverdin, ferrous iron, and carbon monoxide (CO) [36]. These products have a regulated effect in the physiological processes of inflammation, apoptosis and proliferation, oxidative stress, and angiogenesis [37,38]. HO-1 is highly induced and expressed to eliminate ROS produced by cells under stress conditions, such as heat stress, ischemia-reperfusion injury [39,40]. Interestingly, HO-1 has also been reported to affect the synthesis of VEGF through its three products degraded from heme [41]. 

In this study, we investigated the effects of Mel on follicular development. Compared with FSH, which mainly promoted the development of antral follicles, we observed that Mel primarily acted on secondary follicles. Moreover, both Mel and FSH contributed to follicular angiogenesis by increasing the expression of VEGF. These findings may provide new avenues for the artificial regulation of follicular development.

## 2. Results

### 2.1. Melatonin Promoted Secondary Follicles Development and Periphery Angiogenesis, While FSH Remarkably Increased the Number of Antral Follicles and Periphery Angiogenesis

**Figure 1:**
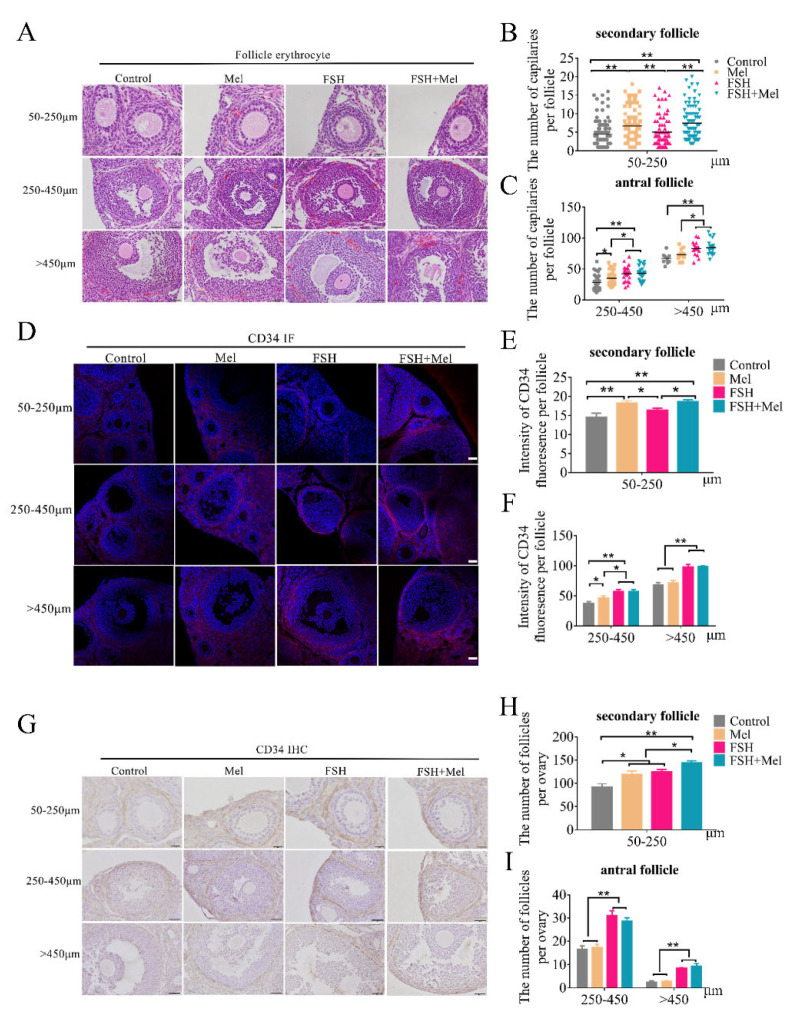
Effect of Mel and FSH on the number of follicles and erythrocytes and the expression of CD34 around follicles in different follicular development stage of mice ovaries. (**A**) Erythrocytes around follicles in different follicular development stage of mice ovaries detected by hematoxylin-eosin (H&E) staining. Scale bar = 20 μm in 50–250 μm follicles, scale bar = 50 μm in above 250 μm follicles. (**B**) Statistical analysis of the amount of erythrocytes in 50–250 μm follicles. (**C**) Statistical analysis of the amount of erythrocytes in above 250 μm follicles. (**D**) The expression of CD34 around follicles in different follicular development stage of mice ovaries detected by immunofluorescence (IF). Scale bar = 50 μm. (**E**) Statistical analysis of the expression of CD34 in 50–250 μm follicles. (**F**) Statistical analysis of the expression of CD34 in above 250 μm. (**G**) The expression of CD34 around follicles in different follicular development stage of mice ovaries detected by immunohistochemistry (IHC). Scale bar = 20 μm in 50–250 μm follicles, scale bar = 50 μm in above 250 μm follicles. (**H**) Statistical analysis of the number of follicles in 50–250 μm follicles. (**I**) Statistical analysis of the number of follicles in above 250 μm follicles. Horizontal bars denote treatments differing *p* < 0.05 *, *p* < 0.01 **.

To assess the effects of melatonin (Mel) on follicle growth, we explored the development of ovarian follicles and the growth of periphery blood vessels using Mel treated mice. In addition, as we all know, the development of follicles is mainly regulated by the pituitary follicle-stimulating hormone (FSH) under physiological conditions. Then we used FSH supplement as a positive reference. The number of ovarian follicles was analyzed in each group after hematoxylin-eosin (H&E) staining. As shown in Figure 1H,I, compared with the control group, the number of 50–250 μm follicles (secondary follicles) in the Mel group increased significantly (*p* < 0.05), while the number of other types of follicles had no significant difference. Consistently, the number of 50–250 μm follicles in the FSH+Mel group increased significantly compared with the FSH group (*p* < 0.05) (Figure 1H), indicating that Mel facilitated secondary follicles development. The number of follicles of 250–450 μm and >450 μm in the FSH and FSH+Mel group increased prominently compared with the control and Mel group (*p* < 0.01) (Figure 1I), suggesting FSH remarkably increased the number of antral follicles. Since angiogenesis is indispensable to the development of follicles, we next investigated whether Mel influence the growth of blood vessels around the developing follicles. The erythrocytes around follicles and the expression of vascular endothelial progenitors CD34 [42] was detected by immunohistochemistry (IHC) and immunofluorescence (IF). As shown in Figure 1A–G, the count of erythrocytes around 50–250 μm follicles in Mel and FSH+Mel group increased significantly compared with the control group (*p* < 0.01), but there was no significant difference in FSH group compared with the control group (*p* > 0.05) (Figure 1A,B). Similarly, the expression of CD34 in 50–250 μm follicles in Mel and FSH+Mel group increased very significantly compared with the control group (*p* < 0.01) and increased significantly compared with FSH group (*p* < 0.05) (Figure 1D,E), showing Mel was conducive to angiogenesis around secondary follicles. In antral follicles (above 250 μm), both the number of erythrocytes and the expression of CD34 increased very significantly in FSH and FSH+Mel group compared with the control group (*p* < 0.01) (Figure 1A,C,D,F), demonstrating FSH stimulated the development of blood vessels. Thus, these findings suggested that Mel and FSH acted in different follicular development period.

### 2.2. Melatonin Enhanced Cell Proliferative Capacity in Ovary

**Figure 2:**
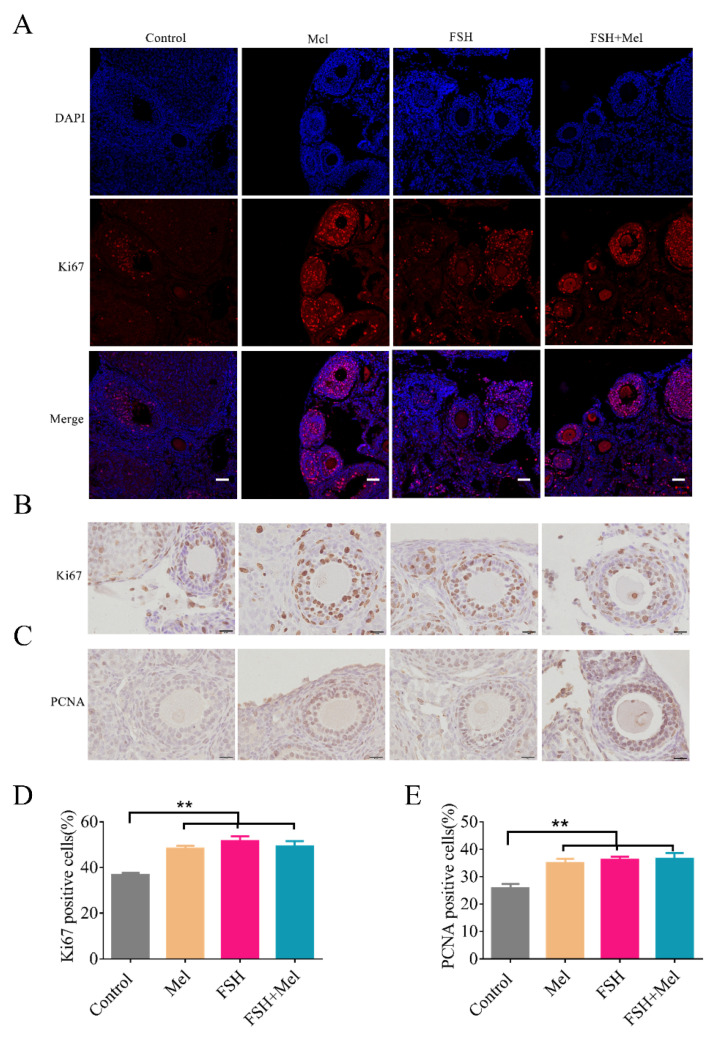
Effect of Mel on the proliferation of ovarian cells. (**A**) Detection of Ki67 expression level by immunofluorescence (IF) (**A**), immunohistochemistry (IHC) (**B**). (**C**) Detection of PCNA expression level by immunohistochemistry (IHC). (**D**) Quantification analysis of the Ki67 expression level. (**E**) Quantification analysis of the PCNA expression level. Horizontal bars denote treatments differing *p* < 0.01 **.

Follicular development relies on cell proliferation in the ovary. Cells proliferation need hormones regulation [43]. Mel and FSH are endogenous hormones exist in follicular fluids [28]. FSH protects mouse granulosa cells from oxidative damage [44] and promotes the proliferation [43]. Whether Mel strengthens cell proliferation in ovary, the protein level of Ki67 and PCNA (proliferating cell nuclear antigen), two proliferation markers [45], were determined by IF and IHC. As shown in Figure 2, the expression of Ki67 in secondary follicles in Mel, FSH and FSH+Mel group was significantly increased in comparison to the control group (*p* < 0.01) (Figure 2A,B,D). The expression of PCNA emerged the analogous result (Figure 2C,E).

### 2.3. Melatonin Raised the Expression of VEGFA in Secondary Follicles, While FSH Conspicuously Enhanced the Expression of VEGFA in Antral Follicles

**Figure 3:**
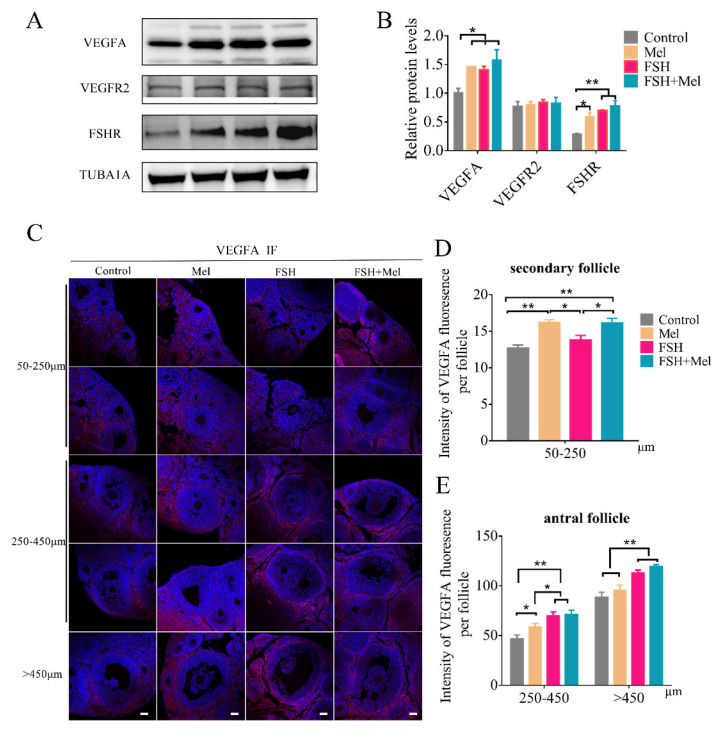
Effect of Mel on the expression of VEGFA. (**A**) Western blotting detection of VEGFA, VEGFR2, FSHR protein expression level in ovary. (**B**) Quantification analysis of the VEGFA, VEGFR2, FSHR protein expression level. (**C**) Immunofluorescence (IF) detection of VEGFA expression level in follicles at all stages. (**D**) Quantification analysis of the VEGFA expression level in 50–250 μm follicles. (**E**) Quantification analysis of the VEGFA expression level in 250–450 μm and >450 μm follicles. Horizontal bars denote treatments differing *p* < 0.05 *, *p* < 0.01 **.

Vascular endothelial growth factor (VEGF), also called VEGFA, as one of the prime candidates regulating ovarian blood vessel formation, acts through one of its two receptors or both [15,46]. To evaluate the effect of Mel on angiogenesis whether through VEGF pathway, the expression of VEGFA and VEGF receptor 2 (VEGFR2) was tested by Western blotting (WB) and immunofluorescence (IF). Results showed that the expression of VEGFA in the ovary was significantly increased in the Mel group, FSH group, and FSH+Mel group compared with the control group (*p* < 0.05) (Figure 3A,B). There was no significant difference between the groups of VEGF receptor 2 (VEGFR2) (Figure 3A,B). Further evidence for a role of VEGF in the regulation of gonadotropin-dependent ovarian follicular angiogenesis and folliculogenesis [15]. Then we measured the protein level of FSH receptor (FSHR). As shown in Figure 3A,B, compared with the control group, the expression of ovarian FSHR in the Mel group was significantly increased (*p* < 0.05), extremely significant increase in FSH group and FSH+Mel group (*p* < 0.01) (Figure 3A,B). To further investigate the effect of Mel on which stage of VEGFA expression, we used IF technology to detected the developing follicles. Results indicated that the expression of VEGFA in the 50–250 μm follicles of the Mel group and FSH+Mel group was significantly increased compared with the control group (*p* < 0.01) (Figure 3C,D). In antral follicles, the expression of VEGFA in FSH group and FSH+Mel group increased very significantly compared with the control group (*p* < 0.01) (Figure 3C,E). Compared with the control group, the expression of VEGFA in 250–450 μm follicles in Mel group increased significantly (*p* < 0.05), but there was no significant difference of >450 μm follicle (Figure 3C,E). These results suggested that Mel mainly increased the expression of VEGFA in secondary follicles, in according with the angiogenesis and CD34 findings. Concomitantly, FSH primarily enhanced the expression of VEGFA in antral follicles.

### 2.4. Melatonin-Regulated Angiogenesis May Not through the HIF-1α/VEGF Signaling Pathway in Secondary Follicles

**Figure 4:**
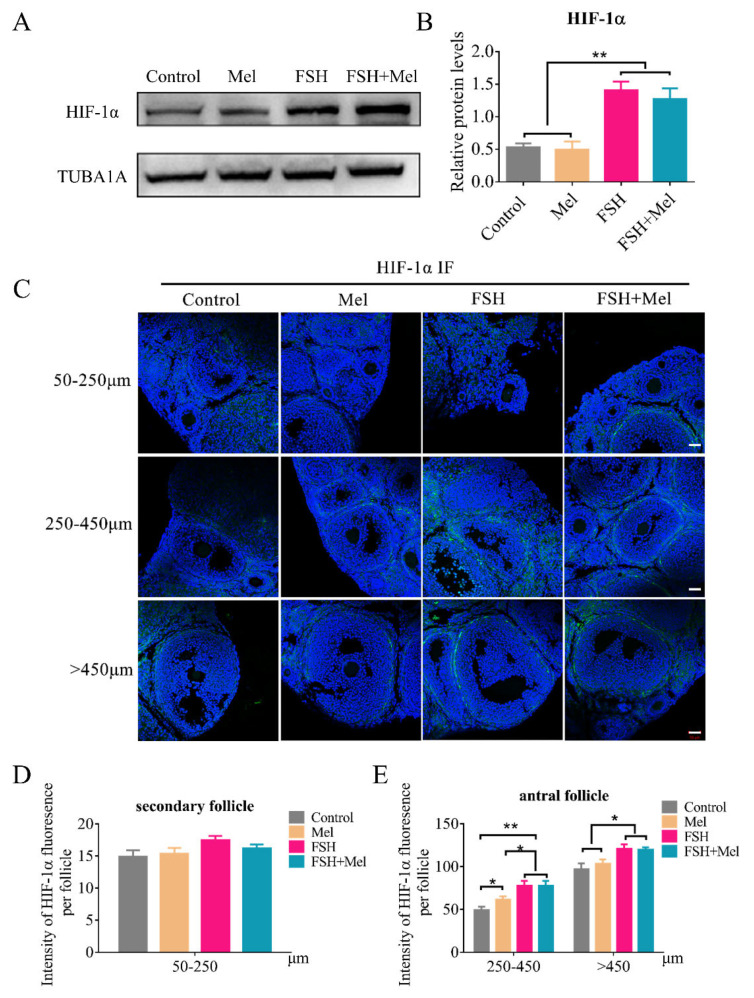
Effect of Mel on the expression of HIF-1α. (**A**) Western blotting detection of HIF-1α protein expression level in ovary. (**B**) Quantification analysis of the HIF-1α protein expression level. (**C**) Immunofluorescence (IF) detection of HIF-1α expression level in follicles at all stages. (**D**) Quantification analysis of the HIF-1α expression level in 50–250 μm follicles. (**E**) Quantification analysis of the VEGFA expression level in 250–450 μm and >450 μm follicles. Horizontal bars denote treatments differing *p* < 0.05 *, *p* < 0.01 **.

As a transcription factor regulating VEGFA, hypoxia-inducible factor 1-alpha (HIF-1α) can be involved in the modulation of angiogenesis. Subsequently, the expression of HIF-1α was determined by Western blotting. As shown in Figure 4A,B, the protein level of HIF-1α in Mel group had no significant difference compared with the control group (Figure 4A,B). Actually, FSH significantly promoted the expression of HIF-1α (Figure 4A,B). Furtherly, the expression of HIF-1α in all stages of follicles was detected by IF technology. It was verified that Mel did not affect the HIF-1α expression level in secondary follicles (50–250 μm follicles) (Figure 4C,D). While we also found that FSH did not influence the expression of HIF-1α in secondary follicles either (Figure 4C,D). In addition, we discovered that the expression of HIF-1α in the FSH group and FSH+Mel group increased significantly compared with the control group around antral follicles (Figure 4C,E), indicating that FSH modulated antral follicular angiogenesis may through the HIF-1α/VEGF signaling pathway.

### 2.5. Melatonin Increased the Expression of Antioxidant Enzymes and Alleviated the Declined Expression of Antioxidant Enzymes Induced by FSH

**Figure 5:**
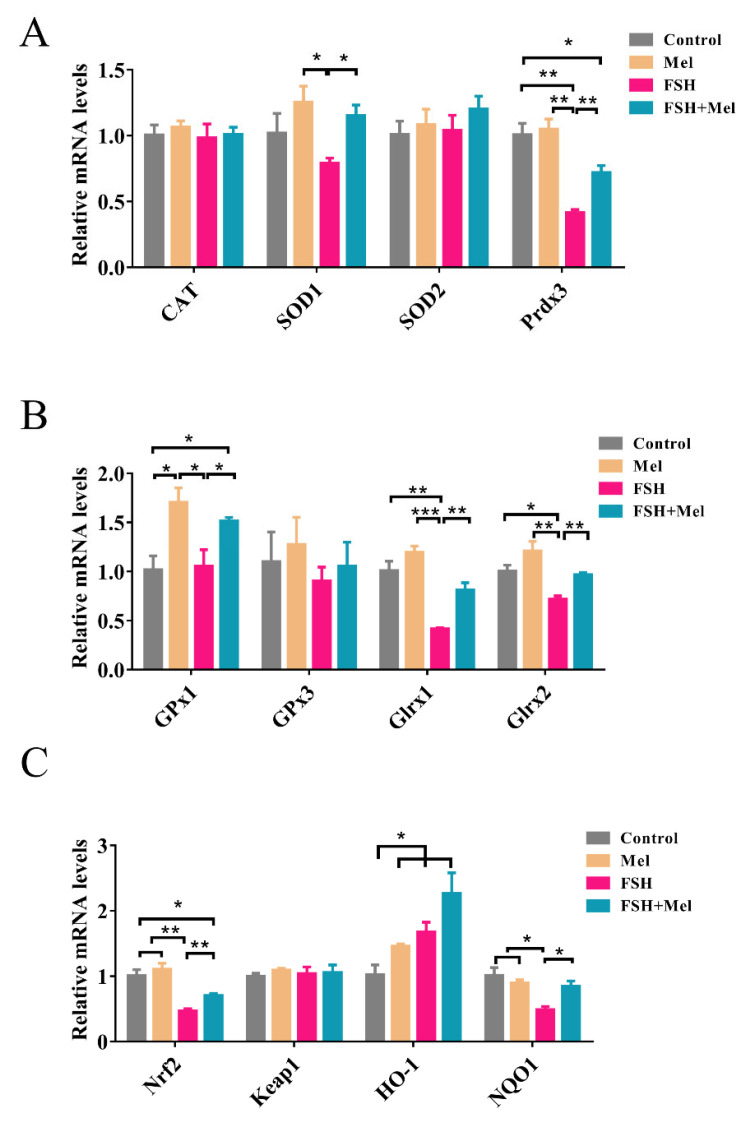
Effect of Mel and FSH on the expression of antioxidant enzymes in mRNA level. (**A**) The mRNA level expression of *CAT* (catalase), *SOD1*, *SOD2* and *Prdx3* in ovary. (**B**) The mRNA level expression of *Gpx1*, *Gpx3*, *Glrx1* and *Glrx2* in ovary. (**C**) The mRNA level expression of *Nrf2*, *Keap1*, *HO-1* and *NQO1* in ovary. Horizontal bars denote treatments differing *p* < 0.05 *, *p* < 0.01 **, *p* < 0.001 ***.

Mel promoted the expression of VEGFA in secondary follicles, but it did not improve HIF-1α expression level, hence, Mel regulated angiogenesis may not through the HIF-1α/VEGF signaling pathway in secondary follicles. Then the mechanism of Mel promoted secondary follicle VEGF expression and angiogenesis need to be further explored. Mel, as a potent antioxidant, can directly act or indirectly function by activating antioxidant enzymes. Subsequently, the expression of antioxidant enzymes in mRNA levels was examined. As shown in Figure 5, the glutathione peroxidase-1 (*Gpx1*) and heme oxygenase-1 (*HO-1*) mRNA expression levels were significantly up-regulated in the Mel group in comparison to the control group (*p* < 0.05) (Figure 5A–C). Besides, we found that the expression of peroxiredoxin-3 (*Prdx3*), glutaredoxin 1 (*Glrx1*), glutaredoxin 2 (*Glrx2*), nuclear factor erythroid 2-related factor 2 (*Nrf2*) and NAD(P)H-quinone oxidoreductase 1 (*NQO1*) in the FSH group was significantly down-regulated (*p* < 0.05) (Figure 5A–C), while only *HO-1* in the FSH group was significantly up-regulated (*p* < 0.05) (Figure 5C) compared with the control group. Furthermore, we discovered that the expression of superoxide dismutase 1 (*SOD1*), *Prdx3*, *Gpx1*, *Glrx1*, *Glrx2*, *Nrf2* and *NQO1* in the FSH+Mel group was conspicuously increased compared with the FSH group (*p* < 0.05) (Figure 5C). In general, while FSH promotes the rapid development of follicles, it will exhaust the antioxidant capacity of ovarian tissue and related antioxidant enzymes. Mel supplement can relieve this situation to a certain extent.

### 2.6. Melatonin Increased the Expression of HO-1 in Secondary Follicles

**Figure 6:**
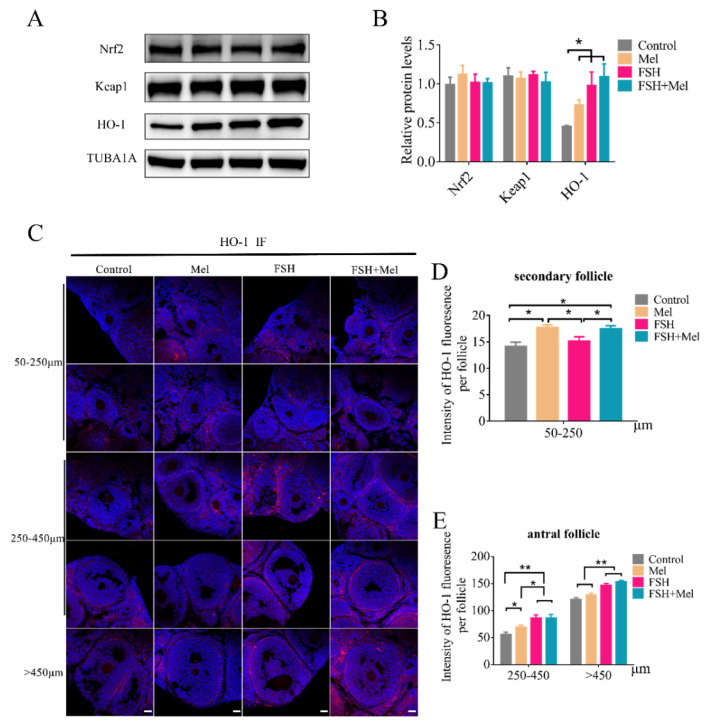
Effect of Mel and FSH on the expression of HO-1, Nrf2 and Keap1. (**A**) Western blotting detection of HO-1, Nrf2 and Keap1 protein expression level in ovary. (**B**) Quantification analysis of the HO-1, Nrf2 and Keap1 protein expression level. (**C**) Immunofluorescence (IF) detection of HO-1 expression level in follicles at all stages. (**D**) Quantification analysis of the HO-1 expression level in 50–250 μm follicles. (**E**) Quantification analysis of the HO-1 expression level in 250–450 μm and >450 μm follicles. Horizontal bars denote treatments differing *p* < 0.05 *, *p* < 0.01 **.

Heme oxygenase-1 (HO-1) was regulated through the nuclear factor erythroid 2-related factor 2 (Nrf2) binding to antioxidant response element (ARE) sequence protecting against oxidative injury and contributing to angiogenesis. Then the expression of HO-1, Nrf2 and Keap1 was determined by Western blotting. As shown in Figure 6A,B, the HO-1 protein level in ovary in the Mel group had a significant increment compared with the control group (*p* < 0.05), consistent with the mRNA result (Figure 5C). However, with the expression of Nrf2 and Keap1 in the ovary, there was no significant difference between the groups (Figure 6A,B). Moreover, the expression of HO-1 in ovary of the FSH group and FSH+Mel group significantly increased compared with the control group (*p* < 0.05) (Figure 6A,B), indicating that Mel and FSH induced increase of HO-1 protein is not carried out through the Nrf2 signaling pathway. Furthermore, the expression of HO-1 in developing follicles was detected by immunofluorescence. As shown in Figure 6C,D, in secondary follicles, compared with the control group, the expression of HO-1 in the Mel and FSH+Mel group was significantly increased (*p* < 0.05), but there was no significant difference in the FSH group (Figure 6C,D). Further, in antral follicles, the expression of HO-1 in the FSH and FSH+Mel group was obviously increased compared with the control group (*p* < 0.01) (Figure 6C,E).

## 3. Discussion

In this manuscript, we provided novel evidence suggesting that Mel specifically stimulated the development of secondary follicles. Compared with Mel, FSH mainly promoted the development of antral follicles as expected. Correspondingly, Mel and FSH acted on the growth of blood vessels in secondary follicles and antral follicles respectively.

Mammalian ovary contains follicles at different stages, including primordial, primary, secondary, and antral follicles. In this study, we found that Mel specifically promoted the development of secondary follicles. This is different with other reports. Jang et al. found that co-treatment with the antioxidant melatonin prevented cisplatin-induced disruption of the follicle reserve, preserving the number of primordial follicles in the ovary, which quantified the various stages of growing follicles, including primordial, primary, secondary, and antral [47]. Yang et al. have shown that administration of melatonin to postnatal mice during the follicle activation phase and the early follicle growth phase, can reduce the number of activated follicles and suppress follicle growth and atresia, respectively [48]. Tamura et al. found that old mice treated with melatonin had significantly more primordial, primary, secondary and antral follicles than the old controls [49]. Our results showed that in prepubertal mice, intraperitoneal injection of Mel markedly increased the number of secondary follicles. Therefore, we concluded that Mel might involve in the early development of ovarian follicles, but the underlying mechanisms remain to be uncovered. 

In the ovary, periodic development of follicles is accompanied by cyclical angiogenesis. Previous researches demonstrated that the formation of an individual capillary network around each follicle is required for follicular growth [50]. Basini et al. reported that melatonin stimulated physiological follicular angiogenesis as evidenced by angiogenesis bioassay using aortic endothelial cell line [51]. In this study, we further revealed that Mel specifically promoted angiogenesis in secondary follicles. Since blood vessel growth is crucial for follicle development, Mel-mediated follicular development might be achieved via stimulating angiogenesis. On the other hand, Mel was reported to prevent primordial follicles loss under stress conditions. Since primordial follicles represent a stage not rely on vascular development, the quantified formation of subsequent secondary follicles might be independent of angiogenesis. 

Vascular endothelial growth factors (VEGFs), a family of potently angiogenic peptides, is ubiquitously expressed in tissues/organs, including ovary. VEGFA is the most abundant form of VEGFs in the ovary [50]. In cow’s ovary, Yang et al. detected VEGFA expression in the granulosa and theca layers of secondary follicles [52]. In addition, VEGFA administration succeeded in stimulating the development of secondary follicles [52]. Our results showed that Mel increased the expression of VEGFA in secondary follicles, indicating a possible involvement of VEGFA in Mel-induced development of secondary follicles. To elucidate the regulatory mechanism of VEGFA expression, we detected the protein level of hypoxia-induced factor 1α (HIF-1α), a potent transcriptional activator of VEGFA. However, the data showed no evident influence of Mel on HIF-1α accumulation, suggesting that Mel-mediated angiogenesis may not depend on the HIF-1α/VEGF signaling pathway in secondary follicles. Unlike Mel, FSH remarkably increased the expression of VEGFA and HIF-1α in antral follicles. Actually, our previous study has reported that FSH acted through HIF-1α to activate VEGF-dependent angiogenesis in antral follicles [53]. Our findings delineated a spatio-temporal-specific set of signal pathways involved in regulating blood vessel growth during follicular development.

Moreover, we found that Mel specifically increased the expression of heme oxygenase-1 (HO-1) in the secondary follicle. Some studies have shown that HO-1 can affect the expression of VEGF through its degradation products of heme. Dulak et al. reported that increased HO-1 expression resulted in an augmented VEGF synthesis in vascular smooth muscle cells under both normoxic and hypoxic conditions [41]. Accordingly, Jozkowicz et al. detected up-regulated VEGF expression after activating HO-1 in human microvascular endothelial cells [54]. Based on these findings, our data suggest that Mel may promote VEGFA by regulating the synthesis of HO-1 in the ovary. However, the mechanism of HO-1 in mediating follicular angiogenesis needs further study.

In summary, this study uncovered a novel role of Mel in orchestrating temporal-specific regulation of follicular development and angiogenesis. Our findings might provide potential avenues for improving reproductive performance. However, the interaction between follicular development and angiogenesis remains to be studied in the future.

## 4. Materials and Methods

### 4.1. Ethics Statement

All experiments and treatments were approved and supervised by the Animal Research Institute Committee of Nanjing Agricultural University (Permit Number: IACUC2020132), China.

### 4.2. Reagents and Antibodies

Melatonin (M5250) was purchased from Sigma-Aldrich (St. Louis, MO, USA). FSH was purchased from Ningbo Second Hormone Factory (Ningbo, China). The relevant information of antibodies against VEGFA, VEGFR2, FSHR, CD34, HIF-1α, Keap1, Nrf2, HO-1, TUBA1A were listed in Table 1. Other chemical agents used in this study were obtained from Sigma-Aldrich (St. Louis, MO, USA), unless instructed otherwise. 

### 4.3. Animals

In this experiment, a total of 40 3-week-old ICR female mice (purchased from Nanjing Medical University) randomly divided into four groups: control group (control), melatonin group (Mel), follicle stimulating hormone group (FSH) and follicle stimulating hormone plus melatonin group (FSH+Mel). They were given a 12/12 h cycle of light/dark every day, accessing to food and water ad libitum. Intraperitoneally injected with 40 mg/kg of Mel or normal saline for 12 days in Mel and the control group, respectively. FSH group and FSH+Mel group were intraperitoneally injected with normal saline and 40 mg/kg of Mel at 20:00 daily for 12 days, while on the 11th day, injected 10 IU of FSH at 8:00 and 20:00. On the 12th day, 5 IU FSH was injected at 8:00 and 20:00 in these two groups. At 8:00 on the 13th day, the mice were anesthetized for blood sampling, and then sacrificed by cervical dislocation, and the corresponding mouse ovaries and ovarian granulosa cells (GCs) were collected. All blood samples were centrifuged at 1500× *g* for 30 min, and the separated serum samples were stored in a refrigerator at −80 °C for later use.

### 4.4. Ovarian Follicle Collection and H&E Staining

The left ovary of mice was fixed with 4% paraformaldehyde and embedded in paraffin. The paraffin blocks of the ovarian tissue were serially sectioned with a thickness of 5 μm. We took one piece of every six and stained with hematoxylin-eosin (H&E). After staining and mounting, we observed and took pictures under a virtual microscope (Olympus, Tokoyo, Japan). Follicle classification was divided into three types according to diameter, 50–250 μm, 250–450 μm, >450 μm. The oocyte nucleus was used as a marker to count the various follicles in each ovary. 

We observed the development of blood vessels around the follicles of different diameters, and counted the number of red blood cells around the follicles after H&E staining. One red blood cell represented a capillary.

### 4.5. Immunohistochemistry (IHC) and Immunofluorescence (IF)

Using immunohistochemistry (IHC) and immunofluorescence (IF), we detected the expression of CD34, VEGFA, HIF-1α and HO-1. Briefly, after the ovarian sections were deparaffinized by xylene and dehydrated by ethanol gradient, they were repaired in a microwave oven with trisodium citrate for 10 min. We placed the slices in 3% hydrogen peroxide solution for 1 h to inactivate endogenous catalase. After blocking with 5% BSA for 1 h, add CD34 antibody (IHC 1:2500, IF 1:250), VEGFA antibody (1:500), HIF-1α (1:500) and HO-1 antibody (1:500) overnight at 4 °C. We then incubated with horseradish peroxidase (HRP)-conjugated secondary antibodies for 1 h at room temperature. The IF section was dripped with DAPI, incubated for 10 min, then mounted with glycerol, and photographed under a confocal laser microscope (Zeiss LSM900, Jena, Germany), using Image J 1.46 software to analyze the fluorescence intensity. The IHC slices were dripped with SABC and incubated for 1 h at room temperature, DAB developed for 1min, and the nuclei were stained with hematoxylin for 10s. After mounted by dehydrated neutral gum, they were observed and photographed under an inverted optical microscope, (Olympus, Tokoyo, Japan).

### 4.6. Western Blotting

We added 300 μL ice-cold RIPA (Radio Immunoprecipitation Assay) Buffer (Beyotime, Shanghai, China) supplemented with 1 mM phenylmethylsulfonyl fluoride (PMSF; Beyotime, Shanghai, China) to the collected ovarian tissue, break the tissue with a crusher, lysed the tissue on ice for 30 min, centrifuged at 4 °C, 12,000× *g* for 15 min, and aspirated the precipitate to obtain soluble protein. We determined its concentration using a BCA Protein Assay Kit (Beyotime, Shanghai, China). Protein denaturation was performed via boiling cell lysates for 10 min in SDS loading buffer (biosharp, Shanghai, China). Equal amounts of proteins were resolved using 12% SDS-PAGE gel and transferred to PVDF membranes (Millipore, Bedford, MA, USA). After they were blocked with 5% BSA for 1 h at room temperature, we put the PVDF membrane into the corresponding diluted antibody VEGFA (1:500), VEGFR2 (1:500), FSHR (1:500), TUBU1A (1:1000), Nrf2 (1:1000), keap1 (1:1000), HO-1 (1:3000) incubated overnight at 4 °C. The membranes were washed three times with TBST buffer and were incubated with the secondary antibody for 1 h at room temperature. Using an ECL Western blotting detection kit (Advansta, CA, USA), we developed a protein band. The band was analyzed by Image J software (version 1.45; National Institutes of Health, Bethesda, MD, USA) and the values for target proteins were normalized to TUBA1A as the endogenous control.

### 4.7. Real-Time Quantitative RT-PCR Analysis

We collected mouse ovary tissue and added 1mL TRIzol reagent (Invitrogen, CA, USA). After crushing the tissue by Ultrasonic Cell Disruptor, total RNA was isolated and was immediately reverse-transcribed using Prime Script™ RT reagent Kit with gDNA Eraser (TaKaRa Bio Inc., Shiga, Japan). The abundance of *CAT*, *Gpx1*, *Gpx3*, *Glrx1*, *Glrx2*, *SOD1*, *SOD2*, *Prdx3*, *Nrf2*, *keap1*, *HO-1* and *NQO1* mRNA molecules was measured by qRT-PCR. β-actin was used as a housekeeping gene. The primers are showed in Table 2. Relative mRNA expression was calculated by the 2^−ΔΔCT^ method. 

### 4.8. Statistical Analyses

All experiments were repeated at least three times. Data were expressed as mean ± S.E.M. Statistical analyses were performed using the univariate analysis of variance (ANOVA) followed by the Student’s *t*-test with SPSS 21.0 statistical software (SPSS, Inc., Chicago, IL, USA). GraphPad Prism version 7.00 (Graphpad Software, San Diego, CA, USA) was used. If data were normality, the Student’s *t*-test was used for data analysis. If data were not normality, they were log10 transformed. If data were still not normally distributed after log transformation, a Dunnett’s T3 test was used. Horizontal bars denote treatments differing *p* < 0.05 *, *p* < 0.01 **, p < 0.0*0*1 ***.

## Figures and Tables

**Table 1:** The information of antibodies used in this research.

Antibody Name	Antibody Number	Corporation	Application
VEGFA	Orb11554	Biorbyt	WB, IF
VEGFR2	Orb11557	Biorbyt	WB
FSHR	Ab75200	Abcam	WB
CD34	Ab81289	Abcam	IF, IHC
HIF-1α	36169S	Cell Signaling Technology	IF
TUBA1A	2125S	Cell Signaling Technology	WB
Nrf2	16396-1	Proteintech	WB
HO-1	66743-1	Proteintech	WB, IF
Keap1	10503-2	Proteintech	WB

**Table 2:** Primers for qRT-PCR.

Genes	Accession No.	Primer Sequence (5′–3′)	Product Size (bp)
*CAT*	NM_009804.2	F:GTCCCTGCTGTCTCACGTTC R:GACATCAGGTCTCTGCGAGG	128
*Gpx1*	NM_008160.6	F:AGTCCACCGTGTATGCCTTCT R:GAGACGCGACATTCTCAATGA	105
*Gpx3*	NM_008161.4	F:CCTTTTAAGCAGTATGCAGGCA R:CAAGCCAAATGGCCCAAGTT	120
*Glrx1*	NM_001360151.1	F:TATAAAAGGGGTGGCAGAGTCCA R:GCCGCCTTGTTGAAAAATCCC	132
*Glrx2*	NM_001038592.1	F:ATCGTCGTTTTGGGGGAAGTC R:GGAACAGTAAGAGCAGGATGTTT	109
*SOD1*	NM_011434.2	F:AACCAGTTGTGTTGTCAGGAC R:CCACCATGTTTCTTAGAGTGAGG	139
*SOD2*	NM_013671.3	F:CAGACCTGCCTTACGACTATGG R:CTCGGTGGCGTTGAGATTGTT	113
*Prdx3*	NM_007452.2	F:GGTTGCTCGTCATGCAAGTG R:CCACAGTATGTCTGTCAAACAGG	100
*Nrf2*	NM_010902.4	F:GAAGCACGCTGAAGGCACAAT R:TTAGGGCCGTTCTGTTTGACA	192
*keap1*	NM_016679.4	F:TCTACGTCCTCGGAGGCTAT R:GCTCAGGTATTCCAAGTGCTTC	199
*HO-1*	NM_010442.2	F:CAGAGCCGTCTCGAGCATA R:CAAATCCTGGGGCATGCTGT	108
*NQO1*	NM_008706.5	F:TGTAGCCAGCCCTAAGGATCT R:GGCTCTTCTCGCCGCCAT	126
*β-Actin*	NM_007393.5	F:TATAAAACCCGGCGGCGCA R:TCATCCATGGCGAACTGGTG	117

## Supplementary Materials

The following are available online at https://www.mdpi.com/article/10.3390/ijms222011262/s1. Figure S1: details of Figure 3A, Figure S2: details of Figure 4A, Figure S3: details of Figure 6A.

## Abbreviations

CAT	catalase
FSH	follicle-stimulating hormone
FSHR	FSH receptor
GnRH	gonadotropin releasing hormone
Glrx1	glutaredoxin 1
Glrx2	glutaredoxin 2
Gpx1	glutathione peroxidase-1
HIF-1	hypoxia-inducible factor-1
HO-1	heme oxygenase-1
H&E	hematoxylin-eosin staining
IF	immunofluorescence
IHC	immunohistochemistry
LH	luteinizing hormone
Mel	melatonin
NQO1	NAD(P)H-quinone oxidoreductase 1
Nrf2	nuclear factor erythroid 2-related factor 2
PCNA	proliferating cell nuclear antigen
Prdx3	peroxiredoxin-3
ROS	reactive oxygen species
SOD	superoxide dismutase
VEGFs	vascular endothelial growth factors
VEGFR2	VEGF receptor 2
WB	Western blotting
