# Supplementary figures and images for: Effect of Exogenous Melatonin on the Development of Mice Ovarian Follicles and Follicular Angiogenesis
Source: Int J Mol Sci. 2021 Oct 19;22(20):11262. doi: 10.3390/ijms222011262 (PMC8540648; doi:10.3390/ijms222011262)

Figure S1

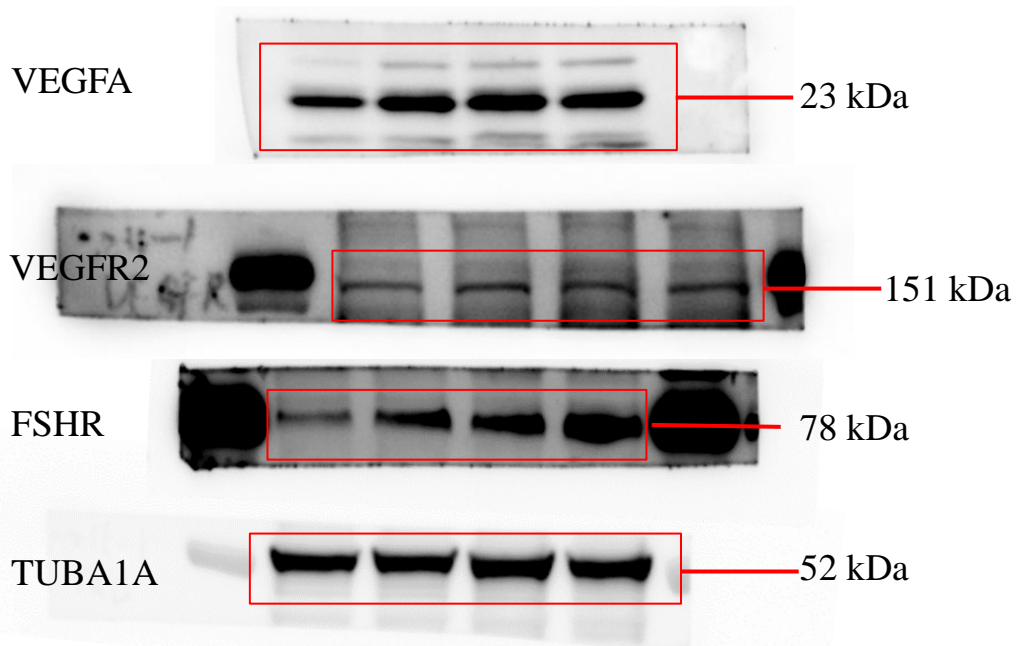

Figure S2

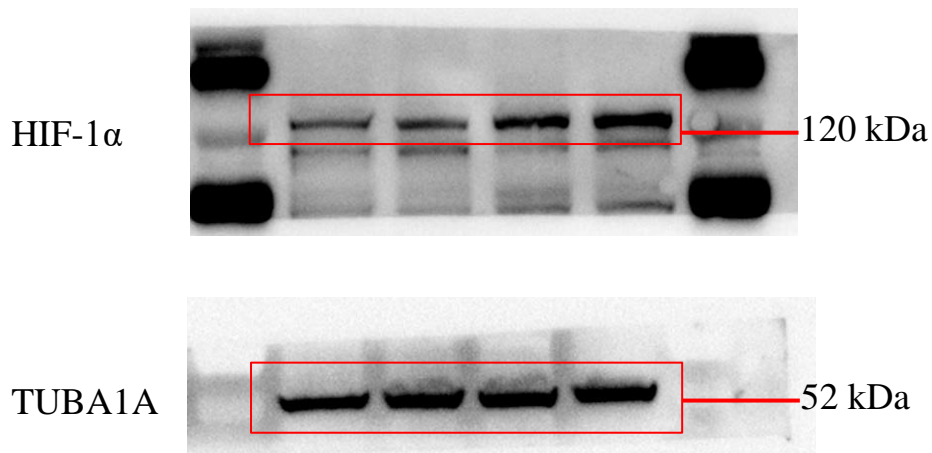

Figure S3

Nrf2

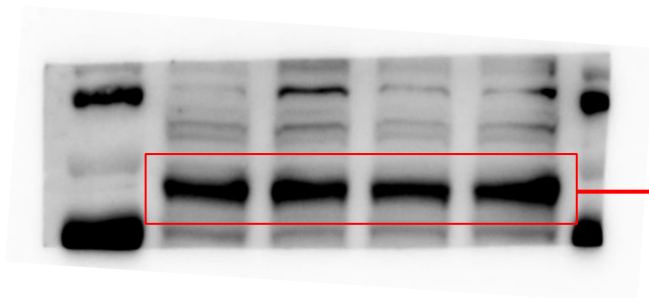

97-100 kDa

Keap1

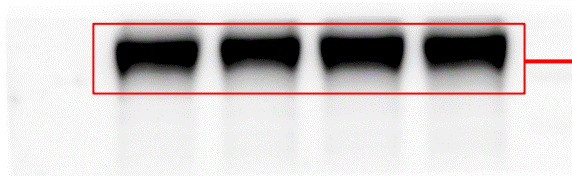

60-64 kDa

HO-1

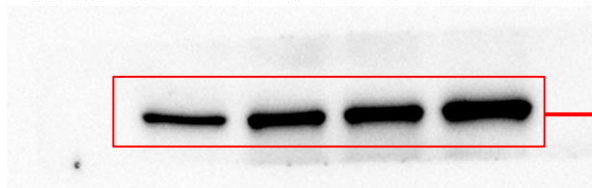

28 kDa

TUBA1A

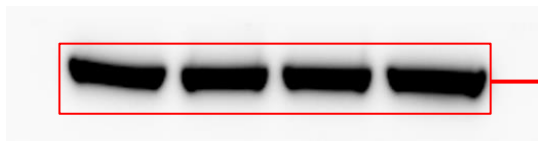

52 kDa

Supplement: Supplementary file 1 [file ijms-22-11262-s001.zip › ijms-1412703-supplementary.pdf]
